# Selective events at individual sites underlie the evolution of monkeypox virus clades

**DOI:** 10.1093/ve/vead031

**Published:** 2023-05-20

**Authors:** Cristian Molteni, Diego Forni, Rachele Cagliani, Federica Arrigoni, Uberto Pozzoli, Luca De Gioia, Manuela Sironi

**Affiliations:** Scientific Institute IRCCS E. MEDEA, Bioinformatics, Via don Luigi Monza, Bosisio Parini 23842, Italy; Scientific Institute IRCCS E. MEDEA, Bioinformatics, Via don Luigi Monza, Bosisio Parini 23842, Italy; Scientific Institute IRCCS E. MEDEA, Bioinformatics, Via don Luigi Monza, Bosisio Parini 23842, Italy; Department of Biotechnology and Biosciences, University of Milan-Bicocca, Piazza della scienza, Milan 20126, Italy; Scientific Institute IRCCS E. MEDEA, Bioinformatics, Via don Luigi Monza, Bosisio Parini 23842, Italy; Department of Biotechnology and Biosciences, University of Milan-Bicocca, Piazza della scienza, Milan 20126, Italy; Scientific Institute IRCCS E. MEDEA, Bioinformatics, Via don Luigi Monza, Bosisio Parini 23842, Italy

**Keywords:** monkeypox virus, positive selection, virus evolution, MPXV clades

## Abstract

In endemic regions (West Africa and the Congo Basin), the genetic diversity of monkeypox virus (MPXV) is geographically structured into two major clades (Clades I and II) that differ in virulence and host associations. Clade IIb is closely related to the B.1 lineage, which is dominating a worldwide outbreak initiated in 2022. Lineage B.1 has however accumulated mutations of unknown significance that most likely result from apolipoprotein B mRNA editing catalytic polypeptide-like 3 (APOBEC3) editing. We applied a population genetics—phylogenetics approach to investigate the evolution of MPXV during historical viral spread in Africa and to infer the distribution of fitness effects. We observed a high preponderance of codons evolving under strong purifying selection, particularly in viral genes involved in morphogenesis and replication or transcription. However, signals of positive selection were also detected and were enriched in genes involved in immunomodulation and/or virulence. In particular, several genes showing evidence of positive selection were found to hijack different steps of the cellular pathway that senses cytosolic DNA. Also, a few selected sites in genes that are not directly involved in immunomodulation are suggestive of antibody escape or other immune-mediated pressures. Because orthopoxvirus host range is primarily determined by the interaction with the host immune system, we suggest that the positive selection signals represent signatures of host adaptation and contribute to the different virulence of Clade I and II MPXVs. We also used the calculated selection coefficients to infer the effects of mutations that define the predominant human MPXV1 (hMPXV1) lineage B.1, as well as the changes that have been accumulating during the worldwide outbreak. Results indicated that a proportion of deleterious mutations were purged from the predominant outbreak lineage, whose spread was not driven by the presence of beneficial changes. Polymorphic mutations with a predicted beneficial effect on fitness are few and have a low frequency. It remains to be determined whether they have any significance for ongoing virus evolution.

## Introduction

Monkeypox virus (MPXV) is the causative agent of mpox. MPXV is a member of the *Orthopoxvirus* genus (family *Poxviridae*), which includes variola virus (VARV, the causative agent of smallpox) and vaccinia virus (VACV, used in the smallpox eradication campaign). Like all poxviruses, MPXV is an enveloped virus with a long (∼190 kb) double-stranded DNA (dsDNA) genome which encodes ∼190 proteins (Hendrickson et al. 2010).

Until recently, mpox was a neglected zoonotic disease occasionally transmitted in an endemic area that ranges from West to Central Africa. In such an area, the diversity of MPXV is genetically structured into two major clades that diverged centuries ago: Clade I is mainly distributed in the Congo Basin and Clade II in West Africa. The latter clade is further divided into two sub-clades broadly corresponding to viruses sampled in Nigeria (IIb) and the west of Nigeria (IIa) (Likos et al. 2005; Berthet et al. 2021; Happi et al. 2022; Forni et al. 2022a). Although the two major clades show only ∼0.5 per cent genomic sequence difference, they are associated with distinct disease presentation. In particular, Clade I viruses are more virulent and cause higher fatality rates than Clade II MPXV (Chen et al. 2005; Likos et al. 2005; Simpson et al. 2020; Bunge et al. 2022).

In the endemic regions, most human mpox cases are zoonotic, although human-to-human transmission was also documented (Yinka-Ogunleye et al. 2019). The animal reservoir(s) of MPXV is presently unknown, but different lines of evidence indicated rodents as the most likely natural hosts of the virus (Doty et al. 2017; Tiee et al. 2018; Forni et al. 2022b). Because MPXV was detected in several rodent species with distinct geographic ranges, it was suggested that the separation of Clades I and II derives both from natural barriers to rodent movements and from adaptation to different reservoir hosts (Forni et al. 2022a,b).

In the last few years, the epidemiology of mpox has been changing. In 2017, the incidence of the disease started to increase in West and Central Africa, both in humans and in primate communities (Rimoin et al. 2010; Sklenovská and Van Ranst 2018; Yinka-Ogunleye et al. 2019; Patrono et al. 2020). In 2018–21, seven human cases reached the UK, the USA, Israel, and Singapore, causing very few secondary transmissions (Reynolds et al. 2006; Ng et al. 2019; Simpson et al. 2020; Adler et al. 2022; Costello et al. 2022; Mauldin et al. 2022; Rao et al. 2022). However, since the beginning of May 2022, a multi-country mpox outbreak has been spreading worldwide. As of this writing (27 March 2023), 86,724 cases have been reported in 110 countries (https://worldhealthorg.shinyapps.io/mpx_global/), and on 23 July 2022, the World Health Organization declared the global monkeypox outbreak a Public Health Emergency of International Concern (Nuzzo, Borio, and Gostin 2022). The epidemiology of the outbreak is characterized by human-to-human transmission, with most infections occurring in young males, who frequently report a previous story of sexually transmitted infections (Adalja and Inglesby 2022; Bragazzi et al. 2022; Thornhill et al. 2022).

Analysis of viral genomes indicated that the outbreak sequences sampled in 2022 cluster together and are phylogenetically related to Clade IIb (Gigante et al. 2022; Isidro et al. 2022). However, genomes in the predominant outbreak lineage (B.1) differ from the Nigerian strains by several single nucleotide substitutions (Gigante et al. 2022; Isidro et al. 2022). Because of the distinctive genomic and epidemiological characteristics, the virus causing the outbreak was re-named human MPXV1 (hMPXV1) (Happi et al. 2022).

The overwhelming majority of hMPXV1 mutations involve GA > AA or TC > TT replacements, suggesting the action of host apolipoprotein B mRNA editing catalytic polypeptide-like 3 (APOBEC3) enzymes (Gigante et al. 2022; Isidro et al. 2022; Forni et al. 2023; O’Toole et al. 2023). Whatever the underlying mutation mechanism(s), it is still unclear whether the observed changes influence viral fitness or modulate viral phenotypes. Also, irrespective of their origin, mutations are expected to be subject to the action of natural selection, which purges the deleterious ones and favors those that are beneficial. Herein, we applied a population genetics–phylogenetics approach to investigate the evolution of MPXV coding genes during historical viral spread in Africa. We also estimated the distribution of fitness effects and related it to the occurrence of mutations in hMPXV1 genomes.

## Results

### Selection patterns in coding regions during MPXV evolution

We first aimed to investigate the selective patterns of MPXV coding genes during the evolutionary history of the two major clades in Africa. To this aim, we retrieved sixty complete or almost-complete genomes (forty-six Clade I and fourteen Clade II), limiting the Clade II sampling to strains collected before 2017 (Supplementary Table S1). This choice was motivated by previous indications that the mutation pattern of MPXV/hMPXV1 started to change since 2017. We next used the gammaMap program (Wilson et al. 2011), which jointly uses intra-species variation and inter-species diversity, to estimate the distribution of fitness effects (i.e. selection coefficients, γ) along coding regions. Thus, our approach differs from previous analyses of positive and negative selections in MPXV genomes (Shan et al. 2022; Guan et al. 2023; Zhan, Zha, and He 2023). In practical terms, *γ*  values can be considered a measure of the fitness consequences of new non-synonymous mutations. The method categorizes selection coefficients into twelve predefined classes ranging from −500 (inviable) to 100 (strongly beneficial). gammaMap allows the detection of fine-scale differences in selective pressures at specific codons and is relatively insensitive to demography and recombination (Wilson et al. 2011).

**Figure 1.**
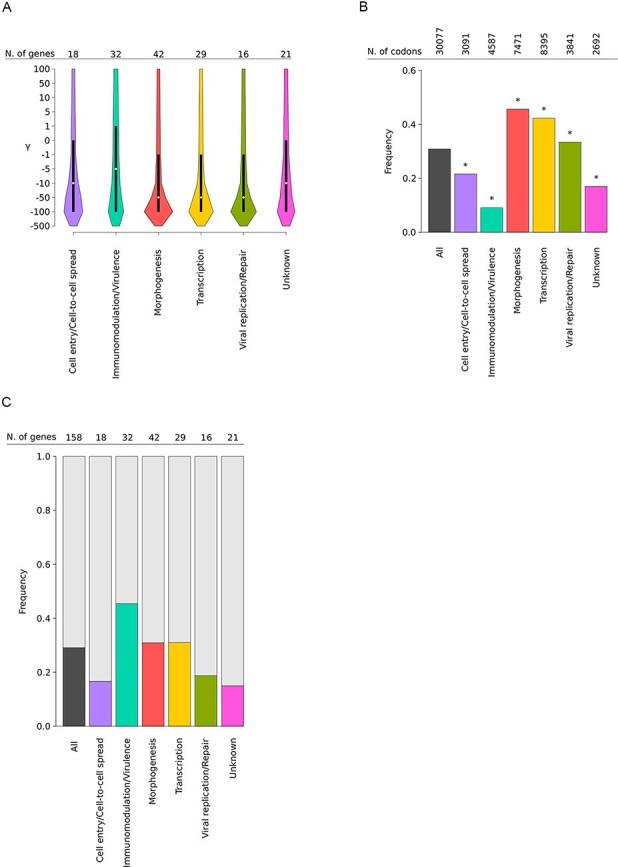
Selective patterns of MPXV genes. **(A)** Violin plots (median, white dot; interquartile range, black bar) of gammaMap selection coefficients (γ) calculated for all codons of the 158 analyzed genes. Genes are grouped and colored based on their functional category (see Supplementary Table S2 for details). The number of genes contributing to each category is also reported. **(B)** Fraction of codons evolving under strong negative selection. The proportion of codons with a cumulative probability > 0.75 of γ ≤ −10 is reported for each functional class (colored bars). The total number of analyzed codons is also reported. Asterisks denote significance at the binomial test (all corrected *P*-values were <0.001). **(C)** Frequency of positively selected genes. Colored bar plots represent the frequency of genes with at least one positively selected codon calculated within each functional category.

gammaMap analysis was performed on 158 genes (see Materials and Methods section, Supplementary Table S2). Overall, we observed a preponderance of codons evolving under strong purifying selection (−500 ≤ *γ* ≤ −10) (Fig. 1A). This was particularly true for viral genes involved in morphogenesis and DNA replication or transcription, which had a high proportion of codons with a cumulative probability >0.75 of *γ* *≤* −10 (Fig. 1B). These genes thus experienced intense constraint (Fig. 1A, Supplementary Table S2). Conversely, genes involved in immunomodulation/virulence and cell entry or spread, as well as those with unknown function, had significantly fewer sites evolving under strong negative selection than the genome average (Fig. 1B).

**Figure 2.**
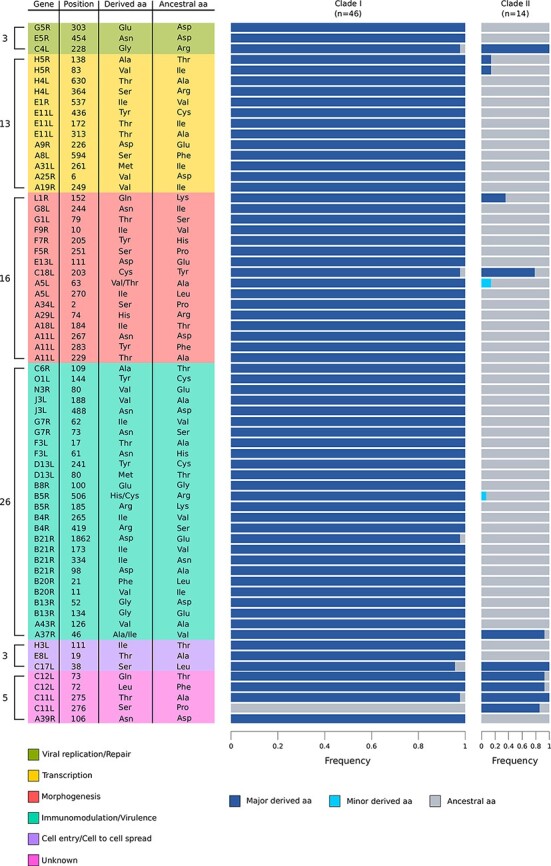
Positively selected sites in the two MPXV clades. The plot shows all the identified positively selected sites, their position (based on the NC_003310 Zaire-96-I-16 strain), and their relative frequency in the two MPXV clades. The number of positively selected sites in each category is also reported. The ancestral amino acid refers to the amino acid coded by the reconstructed outgroup (see Materials and Methods section for details); when more amino acids different from the ancestral were observed in MPXV clades, they are indicated as major and minor derived allele, based on their frequencies.

In all functional classes, a non-negligible fraction of codons displayed selection coefficients consistent with positive selection. To conservatively define signals of positive selection, we estimated codon-wise posterior probabilities for each selection coefficient. We called a codon as positively selected if its cumulative posterior probability of *γ* ≥ 1 was >0.75. Based on the distribution of cumulative probability of *γ* ≥ 1 across all codons, this threshold is conservative (Supplementary Fig. S1). A total of sixty-six sites in forty-six genes (29.1 per cent of the genes we analyzed) were found to be positively selected (i.e. to have at least one positively selected codon) (Supplementary Table S3). Most such sites are biallelic, and in the majority of cases, the selected derived allele is at high frequency in Clade I MPXV (Fig. 2).

Analysis of genes with at least one positively selected site showed that they are differentially distributed in terms of functional classes and those involved in immunomodulation and/or virulence display the highest frequency of positive selection (Fig. 1C). At the codon-wise level, twenty-six sites (39.4 per cent of the total) were found to be located in genes involved in immunomodulation or virulence, a proportion much higher than expected by chance (binomial test, two-sided, *P* = 0.0055) (Fig. 2).

Thus, the overall results of the positive selection scan indicate that the host immune system acted as a major source of pressure during MPXV evolution.

### Analysis of positively selected genes and sites

Because we found that genes with a function in immunomodulation and/or virulence were common targets of positive selection, we focused on this functional category to gain insight into the processes that may have favored MPXV adaptation.

**Figure 3.**
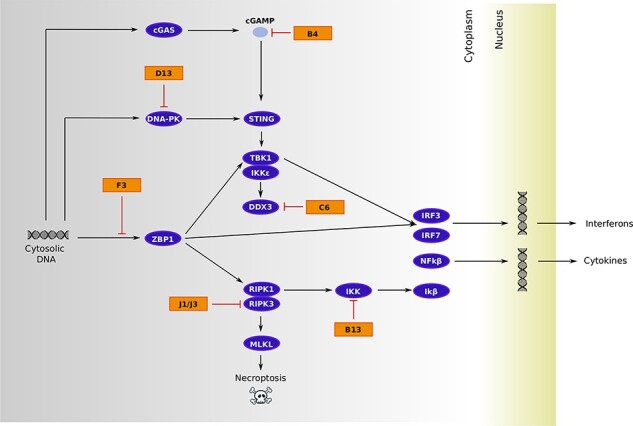
Viral immunomodulatory/virulence proteins that hijack the cytosolic DNA-sensing pathway. The schematic diagram is derived from the Kyoto Encyclopedia of Genes and Genomes pathway repository (map04623) with the addition of relevant interactions: stimulation of the TBK1/IKKe complex by DDX3 (Schröder, Baran, and Bowie 2008), interaction between RIPK3 and MKLK (Sun et al. 2012), role of DNA-PK as a DNA sensor (Ferguson et al. 2012) and its inhibition by proteins belonging to the C4/C16 family (MPXV D13) (Peters et al. 2013; Rivera-Calzada et al. 2022), J1/J3-mediated degradation of RIPK3 (Liu et al. 2021), prevention of Z-DNA sensing by F3 to block ZBP1-mediated signaling (Koehler et al. 2021), inhibition of the IKK complex by B13 (Chen et al. 2008), C6 targeting of DDX3 (Schröder, Baran, and Bowie 2008), and cyclic guanosine monophosphate–adenosine monophosphate (cGAMP) degradation by B4 (Eaglesham et al. 2019). Host proteins are in purple and viral proteins in orange.

Among genes directly involved in immunomodulation, six encode proteins that counteract different steps of the cellular pathway that senses cytosolic dsDNA and activates the production of interferons (IFNs) and cytokines, as well as an inflammatory form of cell death known as necroptosis (Table 1, Fig. 3). Two other virulence genes we identified, A37R and B21R, also impinge on a common pathway and affect T cell activation. Specifically, the VACV-Cop ortholog of A37R (A35R) was shown to decrease the presentation of antigenic peptides by Major Histocompatibility Complex (MHC) Class II molecules, whereas B21R renders T cells unresponsive to stimulation of the T cell receptor by MHC-dependent antigen presentation (Rehm et al. 2010; Alzhanova et al. 2014). Another positively selected gene (N3R, also known as orthopoxvirus MHC Class I–like protein) instead blocks the activation of CD8+ T cells and Natural Killer (NK) cells by binding the NKG2D receptor (Campbell et al. 2007). The remaining immunomodulatory genes encode poorly characterized proteins (Table 1).

**Table 1.** Positively selected genes with a role in immunomodulation and/or virulence.

Gene	OPG	Sites^a^	Features and function	References
A37R	OPG163	V46A/I	A deletion mutant is highly attenuated in the intranasal and intraperitoneal mouse challenge models; inhibits MHC Class II-restricted antigen presentation	Rehm et al. (2010); Roper (2006)
A43R	OPG172	A126V	Putative Type I membrane protein; a deletion mutant produces significantly smaller lesions than the control in the skin of mice	Sood and Moss (2010)
B4R	OPG188	V265I S419R	Poxin; cleaves 2′3′ cGAMP to restrict STING-dependent signaling	Eaglesham et al. (2019)
B5R	OPG189	K185 R R506H/C	ANK repeat- and PRANC domain-containing protein; an ectromelia virus deletion mutant showed decreased spread to organs and was attenuated during mouse infection	Burles et al. (2014)
B8R	OPG192	G100E	Endoplasmic reticulum resident protein; a deletion mutant is attenuated in a murine intradermal model (smaller lesions than the control virus)	Price et al. (2000)
B13 R	OPG200	D52G E134G	Cytosolic virulence factor; inhibits IκB kinase	Chen, Jacobs, and Smith (2006); Chen et al. (2008)
B20R	OPG209	I11V L21F	TNF receptor	Chen et al. (2005)
B21R	OPG210	A98D V173I N334I E1862D	Inhibits TCR-mediated stimulation; deletion reduces virulence in a non-human primate model	Alzhanova et al. (2014)
C6R	OPG044	T109A	Virulence factor that inhibits DDX3	Schröder, Baran, and Bowie (2008)
D13 L	OPG031	T80M C241Y	Inactivates DNA sensing and enhances virulence by binding Ku	Rivera-Calzada et al. (2022)
F3L	OPG065	A17T H61N	Competes with ZBP1 for Z-DNA binding; blocks necroptosis	Koehler et al. (2021)
G7R	OPG091	V62I S73N	Enzyme activity; deletion, or catalytically inactive mutants cause less severe disease in mice than WT viruses	Senkevich et al. (2008)
J3L/J1R^b^	OPG003	A188V D488N	ANK repeat and F-box containing protein; targets RIPK3 for degradation and inhibits necroptosis	Liu et al. (2021)
N3R	OPG016	E80V	Secreted protein; functions as a competitive antagonist of the NKG2D activating receptor	Campbell et al. (2007)
O1L	OPG037	C144Y	ANK repeat containing protein; prevents apoptotic functions of the apoptosome	Ryerson et al. (2017)

Notes: ^a^Amino acid positions refer to MPXV NC_003310 (Zaire-96-I-16).

b Paralog genes located in the ITR.

**Figure 4.**
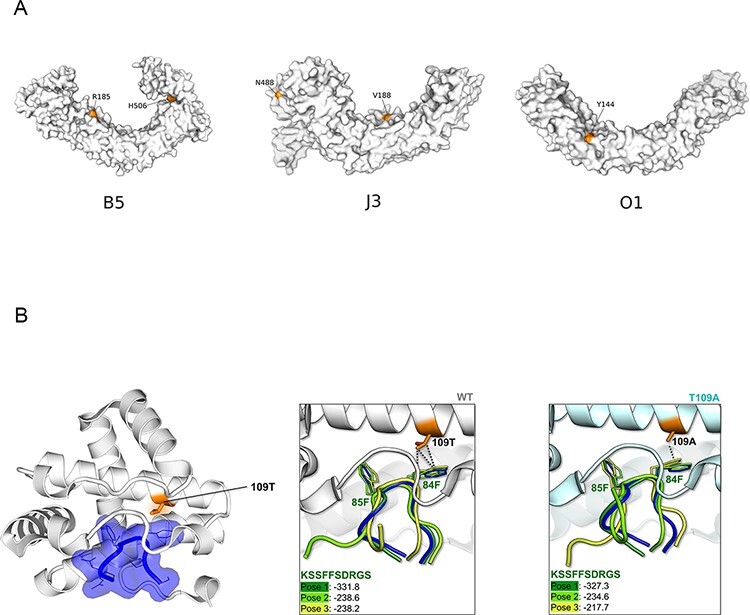
Structural analysis of positively selected sites. **(A)** Structural models of three ANK repeats-containing proteins. Positively selected sites are in orange. **(B)** Crystal (left) and docking (central and right) structure of VACV-Cop K7 with the 81-KSSFFSDRGS-90 peptide of DDX3. In the crystal structure (PDB ID: 3JRV) representation (left), K7 is in white and the peptide in blue. The positively selected site (T109A) is in orange. In the central and right panels, three top-ranked docking poses for 81-KSSFFSDRGS-90 (green shades) to the WT (white, central) and to the T109A mutant (cyan, right) are shown. The peptide, as found in the 3JRV structure, is also reported, as reference (in blue). HPEPDOCK docking scores are also shown.

At the level of protein domains, three of the immunomodulatory/virulence proteins (B5R, J3L/J1R, and O1L) display positively selected sites within ankyrin (ANK) repeats. AlphaFold2 structural models of these proteins showed that the positively selected sites are located on the concave surface, which primarily functions as a protein–protein interaction interface (Ingham, Loubich Facundo, and Dong 2022) (Fig. 4A). Indeed, in the case of J3L/J1R, the ANK domain is sufficient to bind RIPK3, although the structural features of the interaction are unknown (Liu et al. 2021). Conversely, the molecular details of the interaction between the C6R ortholog encoded by VACV-Cop (K7R) and the DDX3 helicase were determined (Oda, Schröder, and Khan 2009). We thus analyzed this interaction as a proof of concept that at least some positively selected sites modulate binding with host proteins.

K7R was crystallized with the 81-KSSFFSDRGS-90 peptide of DDX3, where the tandem phenylalanines are essential for binding (Oda, Schröder, and Khan 2009). K7R and C6R display 95 per cent sequence identity; in the former, position 109 is a threonine, whereas the positively selected 109 site is polymorphic in MPXV, with Clade I viruses all having 109A and Clade II viruses 109T. The 109T residue in K7R is located at the binding interface (Fig. 4A). We thus performed in silico mutagenesis of K7R by introducing the T109A mutation, and we docked the 81-KSSFFSDRGS-90 peptide to the wild-type (WT, 109T) and mutant (109A) proteins. In both cases, the docking poses nicely reproduced the peptide disposition in the crystal structure (Fig. 4B), with the two F residues forming the hydrophobic contacts that drive the binding to K7R. The docking scores were however slightly worse for the mutant, so the T109A substitution may disfavor DDX3 binding. To corroborate the trend of binding affinities upon mutation, the top-ranked docking poses were re-scored by means of the prime molecular mechanics-generalized born surface area (MM-GBSA). Because the calculated absolute binding free energies (ΔG_bind_) may not necessarily correspond to the experimental binding affinities, we will refer to relative ΔG_bind_ values (ΔΔG_bind_). The MM-GBSA ΔΔG_bind_, calculated as ΔΔG_bind_ = ΔG_bind_(WT) − ΔG_bind_(T109A), was −9.7 kcal/mol. So, the T109A substitution is predicted to decrease protein–peptide affinity. Indeed, the T109A mutation is expected to weaken the hydrophobic contacts with residue 84F in the peptide (Fig. 4B).

### Mutations in the hMPXV1-prevalent epidemic lineage

The overwhelming majority of viral genomes sampled during the 2022 outbreak belong to the predominant B.1 lineage and differ from Clade IIb Nigerian strains by several single nucleotide substitutions (Gigante et al. 2022; Isidro et al. 2022). During the outbreak, many additional mutations appeared and are thus polymorphic in the hMPXV1 population. Both B.1 lineage–defining substitutions (fixed changes) and polymorphic mutations are thought to derive from APOBEC-mediated editing (Gigante et al. 2022; Isidro et al. 2022; Forni et al. 2023; O’Toole et al. 2023). Whatever the mutation mechanism, their functional significance and phenotypic consequences remain unknown. We thus reasoned that the codon-wise selection coefficients calculated earlier, albeit relative to viral evolution in the endemic regions, might provide a blueprint to gain insight into the fitness effect of hMPXV1 mutations. We thus retrieved sequence information of 1,850 complete hMPXV1 genomes available in public repositories, and we identified non-synonymous and synonymous mutations that define Lineage B.1, as well as polymorphic changes that have been accumulating during the 2022 outbreak. The overwhelming majority of polymorphic changes are very low frequency (<5 per cent). We next used the gammaMap results to categorize codons into three classes of fitness effects: deleterious (γ < −5), close to neutral (−5 ≤ γ ≤ +5), and beneficial (γ > +5). This choice was motivated by the substantial uncertainty in the probability distribution of similar selection coefficients at each site. Because we aimed to derive information for the largest possible number of codons, we classified them into the three broad classes. Even using this approach, a proportion of codons (39.8 per cent) were not assigned to any class and were not analyzed (see Materials and Methods section).

**Figure 5.**
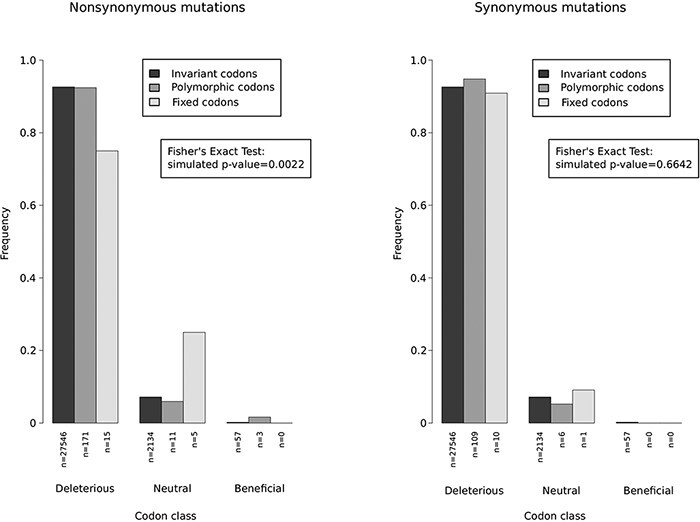
Fitness effect of hMPXV1 mutations. Bar plots showing the frequencies of mutations identified in the B.1 lineage based on the selection coefficient calculated for the Clade I and II strains. Selection coefficients were grouped into three classes: deleterious (γ < −5), close to neutral (−5 ≤ γ ≤ +5), and beneficial (γ > +5). The coefficient distribution of polymorphic (present in at least two B.1 strains) and fixed mutations (present in all B.1 strains) are compared to codons carrying no mutations by a Fisher’s exact test using Monte Carlo simulation (*n* = 10,000) *P*-value.

The distribution of codons carrying no mutations, fixed, or polymorphic changes in the three classes of fitness effects was then analyzed. A deviation from the expected frequencies in codon classes was observed for non-synonymous changes, but not for synonymous substitutions (Fig. 5). Specifically, codons carrying fixed non-synonymous mutations were found to be proportionally less common in the deleterious class compared to codons carrying no or polymorphic non-synonymous changes (Fig. 5). Likewise, codons with fixed non-synonymous mutations were more represented in the close-to-neutral category. No fixed non-synonymous change occurred in codons associated with beneficial effects (Fig. 5). Among codons carrying non-synonymous polymorphic changes, three were associated with a beneficial fitness effect, although the frequency of these mutations is very low (<0.5 per cent).

## Discussion

Most likely, MPXV originated in Africa, possibly West Africa, and, centuries ago, separated into two major lineages (Forni et al. 2022a). These differ in terms of geographic location, virulence, and host association (Forni et al. 2022b). Thus, genetic differences between the two lineages are expected to affect viral phenotypes and, possibly, to represent adaptations to distinct reservoirs. In analogy to what is known for other orthopoxviruses, including VARV, the difference in virulence has mainly been investigated in terms of gene inactivation patterns (Chen et al. 2005). However, no clear evidence has been provided to date that the loss of specific genes can explain differences between the two MPXV lineages (Forni et al. 2022b).

Herein, we only focused on genes that are shared by the two lineages, and by reconstructing their common ancestor, we aimed to determine the selective events that accompanied the emergence and spread of MPXV in West and Central Africa. As is the case of other viruses and cellular organisms alike, we detected a strong prevalence of purifying selection, which is consistent with the notion that most amino acid replacements are deleterious to some degree (Pybus et al. 2007; Wertheim and Kosakovsky Pond 2011; Duchene, Holmes, and Ho 2014; Sironi et al. 2015; Lequime et al. 2016). However, we also detected evidence of positive selection in 29 per cent of the genes we analyzed. Among these, we found a significant over-representation of genes involved in immunomodulation and/or virulence. Six of these encode proteins that play different roles in cytosolic DNA sensing, an essential mechanism by which the host cell detects the presence of the virus and initiates an innate immune response leading to the production of IFNs and cytokines, as well as to the induction of necroptosis. The overall effect of the signal transduction downstream of dsDNA sensing is viral restriction, and many viruses thus encode molecules that hijack distinct steps of the process. As a consequence, the host–virus antagonism turns into a genetic conflict and several proteins of the dsDNA-sensing pathway (e.g. ZBP1, RIPK3, MLKL, STING, and cGAS) are fast evolving in mammals (Hancks et al. 2015; Mozzi et al. 2015; Sironi et al. 2015; Palmer et al. 2021). The signals of positive selection we detected can thus be interpreted within this scenario.

The host–pathogen conflict that plays out during cytosolic DNA sensing must be particularly relevant for poxviruses, which replicate their large dsDNA genomes in the cytoplasm of infected cells. Within this context, we analyzed the effect of the positively selected site in C6R on DDX3 binding. This interaction was selected because structural details were available. In DDX3, the two phenylalanines that play a central role in interaction with the viral protein are conserved across the whole mammalian phylogeny (not shown), suggesting that they are functionally constrained (Oda, Schröder, and Khan 2009). Conversely, in C6R (the ortholog of VACV K7R), the positively selected site is located at the binding interface and our in silico analysis suggests that the T to A change at position 109 decreases affinity for DDX3 binding and possibly fine-tunes IRF3/IRF7 activation (Schröder, Baran, and Bowie 2008). However, the VACV-Cop ortholog of C6R plays additional roles during infection, and we cannot therefore exclude that the positively selected site modulates some other interaction or function (Li, Zhang, and Ke 2017; Teferi et al. 2017).

The orthopoxvirus host range is primarily determined by the interaction with the host immune system rather than by a restriction at the level of cell entry. In this respect, another interesting target of positive selection is B21R. Its directly interacting partner(s) is unknown, but this protein increases virulence by suppressing T cell control of viral dissemination. The action of B21R orthologs from other orthopoxviruses is species-specific (Alzhanova et al. 2014), suggesting that positive selection at this gene contributes to host adaptation.

In terms of gene function, it is also worth noting that, although other genes showing evidence of positive selection are not directly involved in immunomodulation, the underlying selective pressure might still be immune-mediated. For instance, the protein product of the E8L gene (ortholog of VACV-Cop D8L) is an immunogenic envelope protein which binds chondroitin sulfate. The positively selected site (19A/T) is in the epitope bound by the VACV-66 antibody, suggesting that it may represent an immune escape variant (Matho et al. 2018). Likewise, the positively selected site (111T/I) in the H3 protein, which represents a major target of neutralizing antibodies (Davies et al. 2005), flanks the only position (112E) described as a discontinuous B cell epitope in the Immune Epitope Database (https://www.iedb.org/). Finally, mutations in the VACV-Cop ortholog of A25R (A24R) were found to arise during experimental viral evolution and to antagonize the protein kinase R pathway, possibly by allowing evasion from sensors of cytosolic double stranded RNA. The latter is produced during the infection of many viruses, including poxviruses (Weber et al. 2006; Brennan et al. 2015).

The natural host range and reservoir of MPXV are still a matter of debate but seem to include African rodents such as rope squirrels, dormice, and pouched rats (Forni et al. 2022b). Experimental inoculation showed that distinct rodents are differentially susceptible to MPXV-induced pathogenesis. For instance, infection of African rope squirrels causes significant pathology and mortality, whereas Gambian pouched rats suffer less severe disease and lower fatality rates (Falendysz et al. 2015, 2017). From an evolutionary perspective, high pathology in the reservoir host is not advantageous for the virus, although many different factors also play a role. It was thus suggested that, in different geographic locations within the endemic regions, MPXV adapted to diverse hosts (Forni et al. 2022a). Such adaptation might also have entailed a modulation of virulence to establish the prevalence in naive populations. Notably, for the majority of selection signals, the selected derived allele was at high frequency in Clade I viruses. This is possibly secondary to higher power to detect selection, as Clade I genomes were more abundant in public repositories than Clade II sequences (of course with the exclusion of hMPXV1 genomes). However, this observation might also be consistent with an origin of MPXV in West Africa and its subsequent migration in Central Africa, where adaptation to different host species was necessary. In general, the selected sites we identified might contribute to the different virulence of MPXV Clade II versus Clade I.

The gammaMap approach we used for the positive selection scan provides fine-scale information of fitness effects along coding sequences (Wilson et al. 2011). We thus used the selection coefficients associated with the evolutionary events that occurred in the Clade I plus Clade II MPXV populations to infer the effects of mutations that define the predominant hMPXV1 lineage B.1, as well as the changes that have been accumulating during the worldwide outbreak. The results suggested that a proportion of deleterious non-synonymous mutations were purged from the predominant outbreak lineage, whose spread was not primarily driven by the presence of beneficial changes. A similar conclusion was reached by a previous analysis that calculated the expected and observed occurrences of non-synonymous or synonymous changes at APOBEC3 target sites (O’Toole et al. 2023). Conversely, the distribution of non-synonymous substitutions that have been emerging during the outbreak is very similar to that of non-mutated codons and may thus reflect the mutation process, with limited evidence of selection. As a consequence, the large majority of polymorphic non-synonymous mutations are deleterious and are observed in the hMPXV1 population because of genetic drift at transmission or because selection had not enough time to eliminate them. Non-synonymous polymorphic mutations with a predicted beneficial effect on fitness are very few and appear at low frequency. It is currently impossible to determine whether this is because they confer a marginal advantage or because of epidemiological characteristics of the outbreak (overall low-level transmission).

The present study has limitations that need to be taken into account. First, in the analysis of hMPXV1 mutations, we implicitly assumed that the fitness landscape of the virus has remained unchanged since its historical spread in Africa. This clearly represents an over-simplification, as the genetic diversity of MPXV before 2017 primarily reflects evolution in the wild reservoir, whereas the B.1 lineage is thought to have emerged during human infection and its spread was sustained by human-to-human transmission. In fact, the mutation spectra of viruses sampled before and after 2017 differ. It is however conceivable that, for a sizable proportion of codons, selection coefficients are independent of factors such as host or transmission route. For instance, mutations that reduce protein stability or alter enzymatic activity will do so in most (all) hosts or ecological conditions. Second, the division of codons into broad classes is arbitrary in terms of which γ should contribute to each class and of the posterior probability threshold to define classes. Concerning the former, we defined classes so that the representation of γ values is as symmetrical as possible for the three classes. As for the threshold, we set a relatively conservative value that however allowed analysis of the majority of codons. Third, the gammaMap analysis was performed on a relatively small number of complete MPXV genomes with skewed representation of the two major clades. This might have resulted in an overall limited power to detect selective events, with better power for Clade I than for Clade II genomes. Clearly, continued sampling of MPXV/hMPXV1 genomes, both in the historically endemic regions and elsewhere, is expected to improve knowledge of the evolutionary dynamics of this human pathogen.

## Materials and methods

### Viral sequence selection

All complete/almost-complete MPXV genomes belonging to Clades I and II and with detailed coding sequence annotations were retrieved from the National Center for Biotechnology Information (NCBI) database. We then filtered Clade II genomes based on collection date, by removing all strains sampled after 2017; this procedure generated a total of sixty strains divided into forty-six strains from Clade I and fourteen from Clade II (Supplementary Table S1).

In addition, for the reconstruction of the MPXV ancestral gene sequences, we used the two viral species phylogenetically closest to MPXV: horsepox virus (strain MNR-76, NCBI accession number: DQ792504) and cowpox virus strain Finland_2000_MAN (Cowpox VACV-like, NCBI accession number: HQ420893).

### Ortholog detection and ancestral state reconstruction

For all downloaded sequences (sixty MPXV strains sampled before 2017 plus horsepox and cowpox viruses), we performed orthology inference using the OrthoFinder bioinformatic tool (Emms and Kelly 2019). OrthoFinder identifies hierarchical OrthoGroups (OG) as groups of genes that are descended from a common gene in the last common ancestor. An OG can be composed by orthologs and paralogs: in the case of inverted terminal repeat (ITR) genes, we retained only one copy per strain, as they are always identical. Moreover, we considered only genes that are functional in both clades, which are annotated in the NC_003310 reference genome, and are annotated in the 90 per cent of the analyzed strains. OrthoFinder was run using Multiple Alignment using Fast Fourier Transform (MAFFT) as an aligner (Katoh and Standley 2013) and FastTree (Price, Dehal, and Arkin 2010) for tree inferences. The result was a list of 158 orthologous genes, which were used for subsequent analyses (Supplementary Table S2).

In order to reconstruct the ancestral nucleotide sequence of each of the 158 genes, we applied the FastML tool (Ashkenazy et al. 2012). FastML is based on the phylogenetic relationship between homologous sequences, and it requires a multiple sequence alignment and a phylogenetic tree. We generated gene alignments with GUIDANCE2 (Sela et al. 2015), by using MAFFT as an aligner and by masking residues with low alignment scores. The phylogenetic tree was generated using the PhyML software, by applying a general time reversible model (Guindon et al. 2009). FastML estimates the most probable nucleotide for all alignment positions for each internal node of the tree. We retrieved the reconstructed sequence from the internal node that separates the horsepox and the cowpox strains from all the MPXV. We only retained positions (99.6 per cent) for which the most probable ancestral reconstruction had a posterior probability higher than 0.90. Those sequences were then used as out-group sequences for the positive selection analysis.

### Positive selection analysis

Selective events that accompanied the evolution of MPXV were investigated with gammaMap, which uses intra-species variation and inter-species diversity to estimate the distribution of selection coefficient (γ) (Wilson et al. 2011). All 158 OrthoFinder ortholog genes from the sixty MPXV strains sampled before 2017, as well as their corresponding reconstructed ancestral sequences, were analyzed.

gammaMap requires to configure prior distributions for some of the parameters. Thus, we selected weakly informative distributions, meaning improper log-uniform distributions for the transition/transversion ratio (k) and for the branch length (T), a log normal distribution for the neutral mutation rate per site (θ) parameter, and a uniform distribution for the probability of adjacent codons to share the same selection coefficient (p). The frequency distribution of non-stop codons was calculated by merging all the 158 genes.

Two runs of 500,000 iterations were performed with a thinning interval of ten iterations and a burn-in of 50,000. These two runs were merged after checking for their convergence.

Selection coefficient are categorized by gammaMap into twelve predefined classes, ranging from −500 (strongly deleterious) to 100 (strongly beneficial), with zero indicating the neutral class (Wilson et al. 2011). Specifically, the program assigns to each codon a posterior probability for each selection coefficient. Because it is often difficult to infer the relative frequency of similar selection coefficients, individual codons are seldom assigned to one selection class with high reliability (i.e. with high posterior probability). This issue can be overcome by grouping coefficients in larger classes. For instance, for the analysis of MPXV sequences, we were interested in identifying codons that were positively selected during viral spread in Africa. We thus called sites as positively selected if they showed a posterior probability > 0.75 of γ ≥ 1 (in the merged runs).

For the analysis of hMPXV1 mutations, we aimed to infer their fitness consequences. Thus, the twelve selection coefficients were grouped into three categories as follows: beneficial (100, 50, and 10 γ values), close to neutral (5, 1, 0, −1, and −5 γ values), and deleterious (−10, −50, −100, and −500 γ values). Each codon of the 158 analyzed genes was assigned to one of these classes if the sum of the posterior probability of all the corresponding γ values was higher than 0.5.

### hMPXV1 mutations

To analyze the genetic variability of the current outbreak, we retrieved hMPXV1 B.1 high coverage complete genomes from the Global Initiative on Sharing All Influenza Data (GISAID) database. A total of 1,850 sequences were obtained (as of 29 October 2022) (Supplementary Table S4). To identify the mutation spectrum of the B.1 lineage, we applied the Nextclade tool (Aksamentov et al. 2021). All variations of each query sequence were retrieved and assigned to annotated proteins based on a reference sequence. In this study, we used the MPXV-M5312_HM12_Rivers strain (NCBI accession number: NC_063383) as a reference. A total of 1,032 non-synonymous and 553 synonymous unique mutations were identified, and we considered as polymorphic those mutations found in at least two strains (i.e. singletons were discarded to avoid sequencing errors). We considered as fixed mutations those that are present in all B.1 genomes. We then assigned the codons affected by these mutations to one of the three gammaMap categories described earlier.

### Statistical analysis

To evaluate whether genes associated with a specific functional class (Supplementary Table S2) were enriched in positive or negative selection signals, we performed binomial tests under the null hypothesis that all sites have the same probability of positive/negative selection. For instance, in the case of positive selection, such probability is 66/49,963, where sixty-six is the total number of positively selected sites and 49,963 is the total number of codons analyzed by gammaMap. Thus, the null expectation is that positively selected sites are homogeneously distributed in functional classes based on the number of codons in each class. Binomial tests were run for each functional category. Specifically, we considered the number of positively selected sites in each class as the number of successes. The number of trials was set as the number of codons in all genes for that functional class and analyzed in gammaMap, and the probability of success was 66/49,963. Because we analyzed six functional classes, *P*-values were corrected for multiple testing using the false discovery rate method. The same approach was used for negative selection, where the probability was 15,416/49,963.

To compare the distributions of hMPXV1 mutations in the gammaMap classes, we performed Fisher’s exact tests. In particular, we computed *P*-values by running Monte Carlo simulations (*n* = 10,000). All the analyses were run in the R environment.

### Structure prediction and molecular docking

Prediction runs of O1, B5, and J3/J1 3D structures were executed using ColabFold (Mirdita et al. 2022), an easy-to-use software based on AlphaFold2 (Jumper et al. 2021). Default settings were kept for MSA, by using the MMseqs2 clustering module, while template search was disabled and the number of recycles was three. Only the top-ranked model for each protein is presented, which are all characterized by high confidence, with an average local Distance Difference Test  > 85 (except the 1–200 region of B5R, for which the average lDDT is ∼ 40).

Molecular docking between VACV-Cop K7 and DDX3 peptide was performed using HPEPDOCK 2.0, a hierarchical protein–peptide docking algorithm that considers peptide flexibility through an ensemble of conformations, generated by the MODPEP program (Yan, Zhang, and Huang 2017; Zhou et al. 2018a, 2018b). For a more accurate estimation of relative binding free energies upon K7 mutation, a post-docking rescoring of the best HPEPDOCK poses was performed, by means of prime MM-GBSA (Bashford and Case 2000; Das et al. 2009), as implemented in the Schrödinger suite of software (Schrödinger Release 2022-3: Maestro, Schrödinger, LLC, New York, NY, 2021). To do so, the no strain approach was used, according to which both ligand and protein conformations are obtained from the optimized structure of the complex instead of performing distinct optimizations of the three different states (ligand, protein, and complex).

The PyMOL software was used to generate figures (The PyMOL Molecular Graphics System, Version 2.0 Schrödinger, LLC).

## Supplementary Material

vead031_Supp

## Data Availability

The lists of viral strains analyzed in this study and their Accession IDs are provided in Supplementary Tables S1 and S4.
