# Supplementary material for: Selective events at individual sites underlie the evolution of monkeypox virus clades
Source: Virus Evol. 2023 May 20;9(1):vead031. doi: 10.1093/ve/vead031 (PMC10256197; doi:10.1093/ve/vead031)
Supplement: vead031_Supp [file vead031_supp.zip › Supplementary_Table_S1.docx]

**Supplementary Table S1. List of analyzed strains.**

| **NCBI Accession ID** | **Strain Name** | **Clade** | **Country** | **Isolation year** |
| --- | --- | --- | --- | --- |
| DQ011154 | Congo_2003_358 | I | Congo Republic | 2003 |
| DQ011155 | Zaire_1979-005 | I | Democratic Republic of the Congo | 1978 |
| HM172544 | Zaire 1979-005 | I | Democratic Republic of the Congo | 1979 |
| HQ857562 | V79-I-005 | I | Democratic Republic of the Congo | 1979 |
| JX878407 | DRC 06-0950 | I | Democratic Republic of the Congo | 2006 |
| JX878408 | DRC 06-0970 | I | Democratic Republic of the Congo | 2006 |
| JX878409 | DRC 06-0999 | I | Democratic Republic of the Congo | 2006 |
| JX878410 | DRC 06-1070 | I | Democratic Republic of the Congo | 2006 |
| JX878411 | DRC 06-1075 | I | Democratic Republic of the Congo | 2006 |
| JX878412 | DRC 06-1076 | I | Democratic Republic of the Congo | 2006 |
| JX878413 | DRC 07-0045 | I | Democratic Republic of the Congo | 2006 |
| JX878414 | DRC 07-0046 | I | Democratic Republic of the Congo | 2006 |
| JX878415 | DRC 07-0092 | I | Democratic Republic of the Congo | 2006 |
| JX878416 | DRC 07-0093 | I | Democratic Republic of the Congo | 2006 |
| JX878417 | DRC 07-0104 | I | Democratic Republic of the Congo | 2006 |
| JX878418 | DRC 07-0120 | I | Democratic Republic of the Congo | 2007 |
| JX878419 | DRC 07-0275 | I | Democratic Republic of the Congo | 2007 |
| JX878420 | DRC 07-0283 | I | Democratic Republic of the Congo | 2007 |
| JX878421 | DRC 07-0286 | I | Democratic Republic of the Congo | 2007 |
| JX878422 | DRC 07-0287 | I | Democratic Republic of the Congo | 2007 |
| JX878423 | DRC 07-0337 | I | Democratic Republic of the Congo | 2007 |
| JX878424 | DRC 07-0338 | I | Democratic Republic of the Congo | 2007 |
| JX878425 | DRC 07-0354 | I | Democratic Republic of the Congo | 2007 |
| JX878426 | DRC 07-0450 | I | Democratic Republic of the Congo | 2007 |
| JX878427 | DRC 07-0480 | I | Democratic Republic of the Congo | 2007 |
| JX878428 | DRC 07-0514 | I | Democratic Republic of the Congo | 2007 |
| JX878429 | DRC 07-0662 | I | Democratic Republic of the Congo | 2007 |
| KC257459 | Sudan 2005_01 | I | South Sudan | 2005 |
| KC257460 | DRC Yandongi 1985 | I | Democratic Republic of the Congo | 1985 |
| KJ642612 | Ikubi | I | Democratic Republic of the Congo | 1986 |
| KJ642613 | Congo_8 | I | Democratic Republic of the Congo | 1970 |
| KJ642618 | Cameroon-1990 | I | Cameroon | 1990 |
| KJ642619 | Gabon-1988 | I | Gabon | 1988 |
| KP849469 | Boende_DRC_2008 | I | Democratic Republic of the Congo | 2008 |
| KP849471 | Yambuku_DRC_1985 | I | Democratic Republic of the Congo | 1985 |
| MN702444 | A5_contig_SPADES | I | Central African Republic | 2017 |
| MN702445 | A4_contig_SPADES | I | Central African Republic | 2017 |
| MN702446 | 38c_contig_SPADES | I | Central African Republic | 2018 |
| MN702447 | 18_contig_SPADES | I | Central African Republic | 2018 |
| MN702448 | 015c_contig_SPADES | I | Central African Republic | 2018 |
| MN702449 | B2_contig_SPADES | I | Central African Republic | 2016 |
| MN702450 | B1_contig_SPADES | I | Central African Republic | 2016 |
| MN702451 | A6_contig_SPADES | I | Central African Republic | 2017 |
| MN702452 | A2_contig_SPADES | I | Central African Republic | 2010 |
| MN702453 | A1_contig_SPADES | I | Central African Republic | 2001 |
| NC_003310 | Zaire-96-I-16 | I | Democratic Republic of the Congo | 1996 |
| KP849470 | Cote dIvoire_1971 | IIa | Ivory coast | 1971 |
| AY603973 | MPXV-WRAIR7-61; Walter Reed 267 | IIa | NA | 1962 |
| AY741551 | Sierra Leone | IIa | Sierra Leone | 1970 |
| AY753185 | COP-58 | IIa | NA | 1958 |
| DQ011153 | USA_2003_044 | IIa | USA | 2003 |
| DQ011156 | Liberia_1970_184 | IIa | Liberia | 1970 |
| DQ011157 | USA_2003_039 | IIa | USA | 2003 |
| KJ642614 | UTC | IIa | NA | 1965 |
| KJ642616 | PCH | IIa | NA | 1968 |
| MT903346 | MPXV-USA2003_099_Gambian_Rat | IIa | USA | 2003 |
| MT903347 | MPXV-USA2003_099_Dormouse | IIa | USA | 2003 |
| MT903348 | MPXV-USA2003_099_Rope_Squirrel | IIa | USA | 2003 |
| KJ642615 | W-Nigeria | IIb | Nigeria | 1978 |
| KJ642617 | Nigeria-SE-1971 | IIb | Nigeria | 1971 |
