# Supplementary material for: Selective events at individual sites underlie the evolution of monkeypox virus clades
Source: Virus Evol. 2023 May 20;9(1):vead031. doi: 10.1093/ve/vead031 (PMC10256197; doi:10.1093/ve/vead031)
Supplement: vead031_Supp [file vead031_supp.zip › Supplementary_Table_S2.docx]

**Supplementary Table S2. List of genes used for GammaMap analysis.**

| **Gene^a^** | **OPG** | **Vaccinia virus (Copenhagen)^b^** | **Functional Category** |
| --- | --- | --- | --- |
| A10L | OPG135 | A9L | morphogenesis |
| A11L | OPG136 | A10L | morphogenesis |
| A12R | OPG137 | A11R | morphogenesis |
| A13L | OPG138 | A12L | morphogenesis |
| A14L | OPG139 | A13L | morphogenesis |
| A15L | OPG140 | A14L | morphogenesis |
| A16L | OPG142 | A15L | morphogenesis |
| A17L | OPG143 | A16L | cell_entry/cell_to_cell_spread |
| A18L | OPG144 | A17L | morphogenesis |
| A19R | OPG145 | A18R | transcription |
| A1L | OPG126 | A1L | transcription |
| A20L | OPG146 | A19L | morphogenesis |
| A21L | OPG147 | A21L | cell_entry/cell_to_cell_spread |
| A22R | OPG148 | A20R | viral_replication/repair |
| A23R | OPG149 | A22R | viral_replication/repair |
| A24R | OPG150 | A23R | transcription |
| A25R | OPG151 | A24R | transcription |
| A28L | OPG153 | A26L | morphogenesis |
| A29L | OPG154 | A27L | morphogenesis |
| A2L | OPG127 | A2L | transcription |
| A30L | OPG155 | A28L | cell_entry/cell_to_cell_spread |
| A31L | OPG156 | A29L | transcription |
| A32L | OPG157 | A30L | morphogenesis |
| A33R | OPG159 | A31R | unknown |
| A34L | OPG160 | A32L | morphogenesis |
| A35R | OPG161 | A33R | cell_entry/cell_to_cell_spread |
| A36R | OPG162 | A34R | morphogenesis |
| A37R | OPG163 | A35R | Immunomodulation/virulence |
| A38R | OPG164 | A36R | morphogenesis |
| A39R | OPG165 | A37R | unknown |
| A3L | OPG128 | A2.5L | morphogenesis |
| A40L | OPG167 | A38L | Immunomodulation/virulence |
| A41L | OPG170 | A41L | Immunomodulation/virulence |
| A42R | OPG171 | A42R | unknown |
| A43R | OPG172 | A43R | Immunomodulation/virulence |
| A44R | OPG173 | A43.5R | unknown |
| A45L | OPG174 | A44L | Immunomodulation/virulence |
| A46R | OPG175 | A45R | morphogenesis |
| A47R | OPG176 | A46R | unknown |
| A49R | OPG178 | A48R | viral_replication/repair |
| A4L | OPG129 | A3L | morphogenesis |
| A50R | OPG180 | A50R | viral_replication/repair |
| A5L | OPG130 | A4L | morphogenesis |
| A6R | OPG131 | A5R | transcription |
| A7L | OPG132 | A6L | morphogenesis |
| A8L | OPG133 | A7L | transcription |
| A9R | OPG134 | A8R | transcription |
| B11R | OPG198 | B12R | Immunomodulation/virulence |
| B12R | OPG199 | B13R | Immunomodulation/virulence |
| B13R | OPG200 | B15R | Immunomodulation/virulence |
| B16R | OPG204 | B19R | Immunomodulation/virulence |
| B17R | OPG205 | B20R | Immunomodulation/virulence |
| B19R | OPG208 | C12L | Immunomodulation/virulence |
| B20R | OPG209 | C14L | Immunomodulation/virulence |
| B21R | OPG210 | NA | unknown |
| B2R | OPG185 | A56R | cell_entry/cell_to_cell_spread |
| B3R | OPG187 | B1R | viral_replication/repair |
| B4R | OPG188 | B2R-B3R | Immunomodulation/virulence |
| B5R | OPG189 | B4R | Immunomodulation/virulence |
| B6R | OPG190 | B5R | cell_entry/cell_to_cell_spread |
| B8R | OPG192 | B7R | Immunomodulation/virulence |
| B9R | OPG193 | B8R | Immunomodulation/virulence |
| C10L | OPG048 | F4L | viral_replication/repair |
| C11L | OPG049 | F5L | unknown |
| C12L | OPG050 | F6L | unknown |
| C13L | OPG051 | F7L | unknown |
| C14L | OPG052 | F8L | unknown |
| C15L | OPG053 | F9L | cell_entry/cell_to_cell_spread |
| C16L | OPG054 | F10L | morphogenesis |
| C17L | OPG055 | F11L | cell_entry/cell_to_cell_spread |
| C18L | OPG056 | F12L | morphogenesis |
| C19L | OPG057 | F13L | morphogenesis |
| C1L | OPG039 | K1L | Immunomodulation/virulence |
| C20L | OPG058 | F14L | Immunomodulation/virulence |
| C21L | OPG060 | F15L | morphogenesis |
| C22L | OPG061 | F16L | unknown |
| C23R | OPG062 | F17R | morphogenesis |
| C2L | OPG040 | K2L | cell_entry/cell_to_cell_spread |
| C4L | OPG042 | K4L | viral_replication/repair |
| C5L | OPG043 | K5L | unknown |
| C6R | OPG044 | K7R | Immunomodulation/virulence |
| C7L | OPG045 | F1L | unknown |
| C8L | OPG046 | F2L | viral_replication/repair |
| C9L | OPG047 | F3L | unknown |
| D12L | OPG030 | C5L | unknown |
| D13L | OPG031 | C4L | Immunomodulation/virulence |
| D19L | OPG034 | C1L | unknown |
| D1L-N4R | OPG015-OPG015 | NA | Immunomodulation/virulence |
| D8L | OPG024 | NA | unknown |
| D9L | OPG025 | C9L | Immunomodulation/virulence |
| E10R | OPG122 | D10R | transcription |
| E11L | OPG123 | D11L | transcription |
| E12L | OPG124 | D12L | transcription |
| E13L | OPG125 | D13L | morphogenesis |
| E1R | OPG113 | D1R | transcription |
| E2L | OPG114 | D-D2L | morphogenesis |
| E3R | OPG115 | D3R | morphogenesis |
| E4R | OPG116 | D4R | viral_replication/repair |
| E5R | OPG117 | D5R | viral_replication/repair |
| E6R | OPG118 | D6R | transcription |
| E7R | OPG119 | D7R | transcription |
| E8L | OPG120 | D8L | cell_entry/cell_to_cell_spread |
| E9R | OPG121 | D9R | transcription |
| F10L | OPG073 | E11L | transcription |
| F1L | OPG063 | E1L | transcription |
| F2L | OPG064 | E2L | morphogenesis |
| F3L | OPG065 | E3L | Immunomodulation/virulence |
| F4L | OPG066 | E4L | transcription |
| F5R | OPG068 | E6R | morphogenesis |
| F6R | OPG069 | E7R | cell_entry/cell_to_cell_spread |
| F7R | OPG070 | E8R | morphogenesis |
| F8L | OPG071 | E9L | viral_replication/repair |
| F9R | OPG072 | E10R | morphogenesis |
| G10R | OPG094 | G9R | cell_entry/cell_to_cell_spread |
| G1L | OPG085 | G1L | morphogenesis |
| G2L | OPG086 | G3L | unknown |
| G3R | OPG087 | G2R | transcription |
| G4L | OPG088 | G4L | morphogenesis |
| G5R | OPG089 | G5R | viral_replication/repair |
| G6R | OPG090 | G5.5R | transcription |
| G7R | OPG091 | G6R | Immunomodulation/virulence |
| G8L | OPG092 | G7L | morphogenesis |
| G9R | OPG093 | G8R | transcription |
| H1L | OPG106 | H1L | Immunomodulation/virulence |
| H2R | OPG107 | H2R | cell_entry/cell_to_cell_spread |
| H3L | OPG108 | H3L | cell_entry/cell_to_cell_spread |
| H4L | OPG109 | H4L | transcription |
| H5R | OPG110 | H5R | transcription |
| H6R | OPG111 | H6R | viral_replication/repair |
| H7R | OPG112 | H7R | morphogenesis |
| I1L | OPG077 | I1L | morphogenesis |
| I2L | OPG078 | I2L | cell_entry/cell_to_cell_spread |
| I3L | OPG079 | I3L | viral_replication/repair |
| I5L | OPG081 | I5L | Immunomodulation/virulence |
| I6L | OPG082 | I6L | morphogenesis |
| I7L | OPG083 | I7L | morphogenesis |
| I8R | OPG084 | I8R | transcription |
| J1L-J3R | OPG001-OPG001 | C23L | Immunomodulation/virulence |
| J2L-J2R | OPG002-OPG002 | C22L | Immunomodulation/virulence |
| J3L-J1R | OPG003-OPG003 | C19L | Immunomodulation/virulence |
| L1R | OPG100 | J1R | morphogenesis |
| L2R | OPG101 | J2R | viral_replication/repair |
| L3R | OPG102 | J3R | transcription |
| L4R | OPG103 | J4R | transcription |
| L5L | OPG104 | J5L | cell_entry/cell_to_cell_spread |
| L6R | OPG105 | J6R | transcription |
| M1R | OPG095 | L1R | cell_entry/cell_to_cell_spread |
| M2R | OPG096 | L2R | morphogenesis |
| M3L | OPG097 | L3L | transcription |
| M4R | OPG098 | L4R | morphogenesis |
| M5R | OPG099 | L5R | cell_entry/cell_to_cell_spread |
| N3R | OPG016 | NA | Immunomodulation/virulence |
| O1L | OPG037 | M1L | Immunomodulation/virulence |
| O2L | OPG038 | M2L | unknown |
| P1L | OPG035 | N1L | Immunomodulation/virulence |
| P2L | OPG036 | N2L | unknown |
| Q1L | OPG074 | O1L | unknown |
| Q2L | OPG075 | O2L | viral_replication/repair |

^a^Genes name refer to monkeypox virus NC_003310 (Zaire-96-I-16)

^b^NCBI Accession ID M35027
