# Supplementary material for: Selective events at individual sites underlie the evolution of monkeypox virus clades
Source: Virus Evol. 2023 May 20;9(1):vead031. doi: 10.1093/ve/vead031 (PMC10256197; doi:10.1093/ve/vead031)
Supplement: vead031_Supp [file vead031_supp.zip › Supplementary_Table_S3.docx]

**Supplementary Table S3. Positive selection sites identified by gammaMap.**

| **Gene^a^** | **Position^a^** | **Posterior probability** | **Clade I** | **Clade II** | **Clade I ancestral aa frequency** | **Clade II ancestral aa frequency** | **Clade I derived aa frequency** | **Clade II derived aa frequency** |
| --- | --- | --- | --- | --- | --- | --- | --- | --- |
| A11L | 229 | 0.89 | 46-T | 14-A | 0 | 1 | 1 | 0 |
| A11L | 283 | 0.89 | 46-Y | 14-F | 0 | 1 | 1 | 0 |
| A11L | 267 | 0.9 | 46-N | 14-D | 0 | 1 | 1 | 0 |
| A18L | 184 | 0.87 | 46-I | 14-T | 0 | 1 | 1 | 0 |
| A19R | 249 | 0.76 | 46-V | 14-I | 0 | 1 | 1 | 0 |
| A25R | 6 | 0.76 | 46-V | 14-D | 0 | 1 | 1 | 0 |
| A29L | 74 | 0.87 | 45-H | 14-R | 0 | 1 | 1 | 0 |
| A31L | 261 | 0.88 | 46-M | 14-I | 0 | 1 | 1 | 0 |
| A34L | 2 | 0.9 | 46-S | 9-P | 0 | 1 | 1 | 0 |
| A37R | 46 | 0.97 | 46-A | 13-I;1-V | 0 | 0.07 | 1 | 0.93 |
| A39R | 106 | 0.87 | 46-N | 14-D | 0 | 1 | 1 | 0 |
| A43R | 126 | 0.88 | 46-V | 14-A | 0 | 1 | 1 | 0 |
| A5L | 270 | 0.88 | 46-I | 14-L | 0 | 1 | 1 | 0 |
| A5L | 63 | 0.89 | 46-V | 12-A;2-T | 0 | 0.86 | 1 | 0.14 |
| A8L | 594 | 0.81 | 46-S | 14-F | 0 | 1 | 1 | 0 |
| A9R | 226 | 0.88 | 46-D | 14-E | 0 | 1 | 1 | 0 |
| B13R | 134 | 0.89 | 46-G | 14-E | 0 | 1 | 1 | 0 |
| B13R | 52 | 0.89 | 46-G | 14-D | 0 | 1 | 1 | 0 |
| B20R | 11 | 0.9 | 46-V | 14-I | 0 | 1 | 1 | 0 |
| B20R | 21 | 0.9 | 46-F | 14-L | 0 | 1 | 1 | 0 |
| B21R | 98 | 0.75 | 46-D | 14-A | 0 | 1 | 1 | 0 |
| B21R | 334 | 0.75 | 46-I | 14-N | 0 | 1 | 1 | 0 |
| B21R | 173 | 0.76 | 46-I | 14-V | 0 | 1 | 1 | 0 |
| B21R | 1862 | 0.77 | 45-D;1-E | 14-E | 0.02 | 1 | 0.98 | 0 |
| B4R | 419 | 0.82 | 46-R | 14-S | 0 | 1 | 1 | 0 |
| B4R | 265 | 0.84 | 46-I | 14-V | 0 | 1 | 1 | 0 |
| B5R | 185 | 0.88 | 46-R | 14-K | 0 | 1 | 1 | 0 |
| B5R | 506 | 0.89 | 46-H | 1-C;13-R | 0 | 0.93 | 1 | 0.07 |
| B8R | 100 | 0.84 | 46-E | 14-G | 0 | 1 | 1 | 0 |
| C11L | 276 | 0.85 | 45-P | 2-P;12-S | 1 | 0.14 | 0 | 0.86 |
| C11L | 275 | 0.88 | 1-A;44-T | 14-T | 0.02 | 0 | 0.98 | 1 |
| C12L | 72 | 1 | 45-L | 1-F;13-L | 0 | 0.07 | 1 | 0.93 |
| C12L | 73 | 1 | 45-Q | 13-Q;1-T | 0 | 0.07 | 1 | 0.93 |
| C17L | 38 | 0.96 | 2-L;44-S | 14-S | 0.04 | 0 | 0.96 | 1 |
| C18L | 203 | 0.92 | 45-C;1-Y | 11-C;3-Y | 0.02 | 0.21 | 0.98 | 0.79 |
| C4L | 228 | 0.98 | 45-G;1-R | 14-G | 0.02 | 0 | 0.98 | 1 |
| C6R | 109 | 0.87 | 46-A | 14-T | 0 | 1 | 1 | 0 |
| D13L | 80 | 0.88 | 46-M | 14-T | 0 | 1 | 1 | 0 |
| D13L | 241 | 0.89 | 46-Y | 14-C | 0 | 1 | 1 | 0 |
| E11L | 313 | 0.9 | 46-T | 14-A | 0 | 1 | 1 | 0 |
| E11L | 172 | 0.9 | 46-T | 14-I | 0 | 1 | 1 | 0 |
| E11L | 436 | 0.9 | 46-Y | 14-C | 0 | 1 | 1 | 0 |
| E13L | 111 | 0.85 | 46-D | 14-E | 0 | 1 | 1 | 0 |
| E1R | 537 | 0.77 | 46-I | 14-V | 0 | 1 | 1 | 0 |
| E5R | 454 | 0.78 | 46-N | 14-D | 0 | 1 | 1 | 0 |
| E8L | 19 | 0.89 | 46-T | 14-A | 0 | 1 | 1 | 0 |
| F3L | 61 | 0.86 | 46-N | 14-H | 0 | 1 | 1 | 0 |
| F3L | 17 | 0.88 | 46-T | 14-A | 0 | 1 | 1 | 0 |
| F5R | 251 | 0.86 | 45-S | 14-P | 0 | 1 | 1 | 0 |
| F7R | 205 | 0.87 | 46-Y | 14-H | 0 | 1 | 1 | 0 |
| F9R | 10 | 0.86 | 46-I | 14-V | 0 | 1 | 1 | 0 |
| G1L | 79 | 0.89 | 46-T | 14-S | 0 | 1 | 1 | 0 |
| G5R | 303 | 0.87 | 46-E | 14-D | 0 | 1 | 1 | 0 |
| G7R | 73 | 0.89 | 46-N | 14-S | 0 | 1 | 1 | 0 |
| G7R | 62 | 0.89 | 46-I | 14-V | 0 | 1 | 1 | 0 |
| G8L | 244 | 0.86 | 46-N | 14-I | 0 | 1 | 1 | 0 |
| H3L | 111 | 0.79 | 46-I | 14-T | 0 | 1 | 1 | 0 |
| H4L | 364 | 0.77 | 46-S | 14-R | 0 | 1 | 1 | 0 |
| H4L | 630 | 0.82 | 46-T | 14-A | 0 | 1 | 1 | 0 |
| H5R | 83 | 0.9 | 46-V | 12-I;2-V | 0 | 0.86 | 1 | 0.14 |
| H5R | 138 | 0.91 | 46-A | 2-A;12-T | 0 | 0.86 | 1 | 0.14 |
| J3L | 488 | 0.88 | 39-N | 14-D | 0 | 1 | 1 | 0 |
| J3L | 188 | 0.88 | 39-V | 14-A | 0 | 1 | 1 | 0 |
| L1R | 152 | 0.9 | 46-Q | 9-K;5-Q | 0 | 0.64 | 1 | 0.36 |
| N3R | 80 | 0.83 | 37-V | 14-E | 0 | 1 | 1 | 0 |
| O1L | 144 | 0.89 | 46-Y | 14-C | 0 | 1 | 1 | 0 |

aGenes names and amino acid positios refer to monkeypox virus NC_003310 (Zaire-96-I-16).
