# Supplementary figures and images for: Selective events at individual sites underlie the evolution of monkeypox virus clades
Source: Virus Evol. 2023 May 20;9(1):vead031. doi: 10.1093/ve/vead031 (PMC10256197; doi:10.1093/ve/vead031)

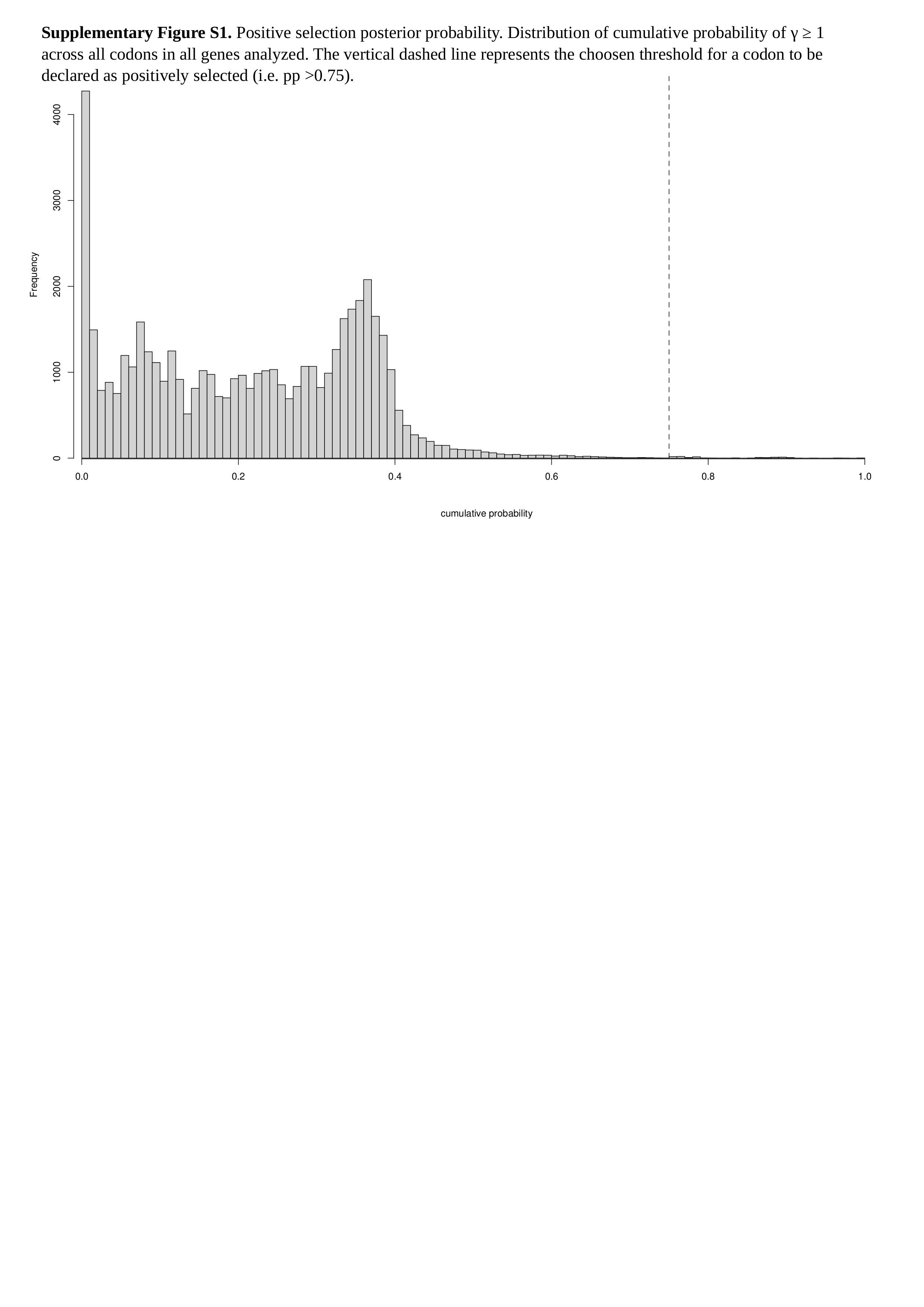

Supplement: vead031_Supp [file vead031_supp.zip › suppl_data/Supplementary_figure_S1.tiff]
